# Urological Applications of Novel Hyaluronic Acid/Alginate Gel to Protect Nerves: A Single-Surgeon Experience

**DOI:** 10.7759/cureus.79156

**Published:** 2025-02-17

**Authors:** Dung Pham, Bryan Pham, Mitchell K Ng

**Affiliations:** 1 Urology, Children’s Memorial Hermann Hospital, Houston, USA; 2 Orthopedic Surgery, Maimonides Medical Center, Brooklyn, USA

**Keywords:** chordee, hidden penis, hyaluronic acid, nerve tethering, orchiopexy, post-operative adhesions, versawrap nerve protector

## Abstract

Background

Tissue gliding and mobility are crucial for the success of penile procedures, as postoperative adhesions can lead to painful tethering of tissues, including nerves. While most commercially available options are not designed to reduce friction and/or often have inconsistent bioresorption, a novel hyaluronic acid/alginate hydrogel represents a significant advancement in reducing postoperative tissue adhesions. This study aimed to evaluate its intra-operative application in pediatric and adult urologic surgeries with associated indications and postoperative outcomes (re-admission, re-operation, complications).

Methods

A retrospective review of 142 male patients undergoing urologic procedures by a single surgeon between October 1, 2022, and October 1, 2023, was conducted after IRB approval. The hydrogel was applied intra-operatively at the surgical site prior to closure. Data on demographics, preoperative diagnoses, and postoperative outcomes were extracted from a prospectively maintained registry and verified through chart review. Diagnoses included 376 conditions such as penoscrotal webbing, hidden penis, and phimosis. Outcomes, including complications, readmissions, and reoperations, were evaluated over an eight-week follow-up period. Descriptive statistics summarized the data and visual tools were used for analysis.

Results

The cohort comprised 142 male patients, predominantly pediatric (93.7%), with the majority (60.0%) under 1 year of age. A total of 376 urologic diagnoses were recorded, with the most frequent being penoscrotal webbing (94 cases, 25.0%), hidden penis (73 cases, 19.4%), and phimosis of the penis (61 cases, 16.2%). Less common diagnoses included hypospadias (6 cases, 1.6%) and various rare conditions with a frequency of 0.27%. Postoperative outcomes showed no complications, re-admissions, or re-operations among the entire patient cohort.

Conclusion

Intra-operative application of a hyaluronic acid/alginate hydrogel in pediatric and adult urologic procedures demonstrates its safety and potential utility in minimizing postoperative tissue adhesions and complications. Further study is warranted to confirm these findings and explore additional applications of this material.

## Introduction

Postoperative soft tissue tethering is a pervasive challenge in many surgical disciplines, particularly in urologic and reconstructive procedures involving delicate anatomical structures like the penile shaft [1]. This tethering is caused by the formation of fibrous tissue bands during healing, joining adjacent tissues that should otherwise remain separate [2]. This pathologic result can lead to a wide array of complications such as pain, restricted tissue mobility, and, in severe cases, significant functional and aesthetic impairments [1]. For example, tethering (adhesions) following circumcision, one of the most commonly performed surgical procedures worldwide, occurs with an estimated incidence of 15%, often resulting in complications such as penile curvature, pain, and skin tethering [3]. Similarly, adhesions can interfere with the outcomes of surgeries addressing hidden penis, chordee, and other urologic conditions, contributing to issues like erectile dysfunction, penile shortening, and psychological distress [2,4].

In penile surgeries, tissue mobility is essential for achieving optimal outcomes. Unwanted postoperative tethering can significantly impair the success of procedures by restricting the natural gliding motion required for nerve and therefore penile function [5]. This tethering can involve not only the nerves but also surrounding soft tissues, leading to reduced sensitivity, numbness, and in some cases, urethral strictures that cause urinary difficulties and increase the risk of infection [6]. Additionally, severe cases may require revision surgeries, compounding patient morbidity and healthcare costs [5,6].

Over the decades, numerous products have been introduced to address this pervasive issue. While some products have shown promise, many have inherent limitations [7,8]. Animal- and human-derived tissue-based products, such as amniotic membrane allografts and collagen-based anti-adhesion implants, have been associated with inconsistent bioresorption [8], foreign body responses, and, in some cases, an exacerbation of inflammatory processes [9]. For example, studies on collagen-based implants have reported incomplete bio-resorption even weeks after surgery, potentially requiring surgical removal [9]. Similarly, a case series involving the use of amniotic membrane allografts in tendon repairs was prematurely terminated due to complications such as tendon rupture and stiffness in half of the cases [8].

To address these challenges, a novel plant-based hydrogel composed of hyaluronic acid and alginate has emerged as a potential breakthrough, allowing soft tissue gliding and protecting peripheral nerve tissues from unwanted tethering [10]. This hydrogel offers several advantages over existing products. Hyaluronic acid (HA) and alginate are widely recognized for their anti-inflammatory, healing, and seamless bioresorption properties [11]. Unlike animal- or human-derived products, this hydrogel is entirely non-immunogenic, eliminating the risk of disease transmission or immune reactions [12]. Its mechanism of action involves immobilizing water on its surface to facilitate tissue gliding and to reduce friction while its hydrophilic but non-swelling nature allows for the diffusion of oxygen, growth factors, and nutrients to underlying tissues without constriction of the protected nerve.

This bioresorbable, translucent, sutureless, novel HA/alginate hydrogel has been effectively employed in a variety of soft tissue settings, including tendons, ligaments, skeletal muscle, and peripheral nerves. The hydrogel is widely applicable across numerous specialties, including but not limited to: plastic surgery, hand surgery (e.g., digital nerve repairs, flexor tendon repairs, cubital tunnel release, microvascular procedures involving tendon and/or nerve, and revision surgeries) [13], spine surgery (e.g., foraminotomy, laminectomy, discectomy) [12], and foot and ankle surgery (e.g., Achilles tendon repair, peroneal tendon reconstruction, ganglion cyst excision, tarsal tunnel release, and ankle arthroscopy) [14]. Its application in penile procedures, however, is less well-documented, despite its significant potential to address the unique challenges posed by these surgeries. By protecting nerves, minimizing tethering, and enabling tissue gliding, this hydrogel may improve functional and aesthetic outcomes while reducing the risk of complications.

This study evaluates the intra-operative use of this HA/alginate hydrogel in pediatric and adult urologic surgeries to protect peripheral nerves, allow tissue gliding, prevent postoperative tethering, and improve surgical outcomes. Specifically, this study aimed to evaluate its intra-operative application in both pediatric and adult urologic surgeries, identifying 1) associated indications for use; 2) associated patient demographics, and 3) postoperative outcomes (re-admission, re-operation, and complications).

## Materials and methods

Study population

After receiving institutional review board (IRB) exemption status, a retrospective review was performed by the first author (DP) to identify patients who underwent various urologic procedures that involved the intra-operative use of a novel hyaluronic acid/alginate gel, VersaWrap Nerve Protector (VersaWrap, Alafair Biosciences, Austin, Texas). VersaWrap comes in the form of an ultra-thin plant-based hydrogel sheet or gel that is believed to facilitate tissue gliding by immobilizing water on its surface, minimizing tissue friction, and decreasing postoperative tethering and pain. VersaWrap has been used in a variety of soft tissue environments (tendons, ligaments, skeletal muscle, peripheral nerves) across different types of surgical procedures to protect tendons and peripheral nerves from postoperative tethering. VersaWrap was applied as a gel intra-operatively around the surgical site and underneath the dartos fascia after surgical exposure prior to closure and then manipulated to ensure appropriate coverage around the localized site of interest (e.g. around the neurovascular bundle and surrounding tissues such as the penile shaft, anchor/plication sutures, or penile skin).

A prospectively generated registry of patients was utilized, with a chart review performed for each patient to verify the inclusion criteria for this study. Data were verified by a full-chart review of the electronic medical record (EMR). This review yielded 142 male patients with a total of 376 associated urologic diagnoses, most of whom were <1 year old. Inclusion criteria included all urologic cases performed from October 1, 2022, to October 1, 2023, with the application of a hyaluronic acid/alginate hydrogel, VersaWrap Nerve Protector (VersaWrap). Exclusion criteria included patients with no postoperative follow-up and/or who did not have intra-operative application of the Versawrap Nerve Protector.

Outcome measures

Baseline patient demographics (age, sex) were recorded, along with preoperative urologic diagnosis. Diagnoses included the following: abscess of a corpus cavernosum and penis, chordee, congenital urinary meatus structure, dysuria, hidden penis, hydronephrosis, hydrocele in adults, hypospadias, hernia, penile adhesions, penile torsion, penoscrotal webbing, Peyronie's disease, phimosis of penis, retractile penis, retractile testis, scrotal pain, second stage of orchidopexy, split urinary stream, testicular mass, undescended testicle, and webbed penis. Postoperative outcomes were measured through a number of re-admissions, re-operations, and complications, which were reviewed and assessed through clinic visits with up to eight weeks of postoperative follow-up. Patients of the primary surgeon (DP) received standard postoperative prescriptions for pain management, oxycodone hydrochloride 5 mg/5 mL, with a small portion of older patients (13) receiving hydrocodone-acetaminophen tablet 5-325 mg.

Statistical analysis

Baseline patient demographics, preoperative diagnoses, application of HA/alginate gel, and associated postoperative outcomes were tabulated into Microsoft Excel (Microsoft Corporation, Redmond, WA). Data were sorted by frequency of type of preoperative urologic diagnosis and by patient demographic information. The respective prevalences of underlying urologic diagnoses were calculated, and a pie chart was generated to represent the data graphically. The age distribution of patients was organized into histograms to display the appropriate frequency distribution. Descriptive statistics were used to summarize the patient cohort’s demographic distribution, calculating arithmetic mean, mode, and range of appropriate continuous variables.

## Results

A total of 142 male patients were included in this study (Table 1).

**Table 1:** Demographic data

Age	
Age Mean (in years)	5.92
Age Median (in years)	0.00
Age Range (in years)	0 to 64
Gender	
Male	142 (100%)
Female	0 (0%)

**Figure 1:**
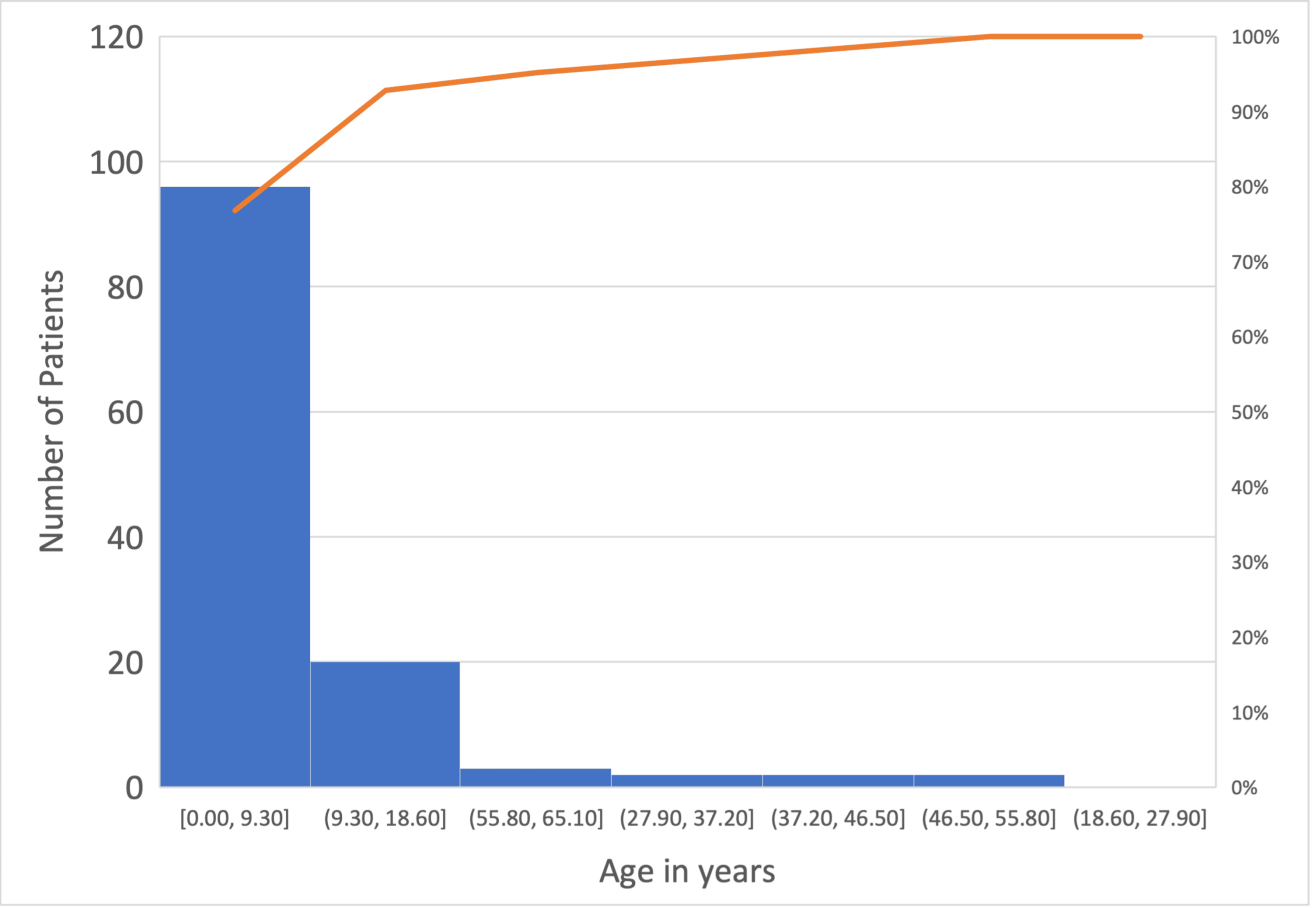
Age distribution of hyaluronic acid/alginate gel application intra-operatively Orange line: overall proportion of cases from 0 years up to and including that age bracket

The majority of patients were pediatric <18 years of age (133/142, or 93.7%), among whom most (85/142, or 60.0%) were under 1 year of age (Figure 1).

Associated urologic diagnoses

A total of 376 underlying urologic diagnoses were included in this study. The underlying urologic diagnoses, ranked by number and frequency, include (from greatest to least): penoscrotal webbing (94 cases, 25.0%), hidden penis (73 cases, 19.4%), phimosis of penis (61 cases, 16.2%), chordee (47 cases, 12.5%), penile torsion (37 cases, 9.8%), penile adhesions (16 cases, 4.3%), hernia (15 cases, 4.0%), undescended testicle (7 cases, 1.9%), hypospadias (6 cases, 1.6%), hydrocele in adults (3 cases, 0.80%), split urinary stream (3 cases, 0.80%), dysuria (2 cases, 0.53%), Peyronie’s disease (2 cases, 0.53%), retractile testis (2 cases, 0.72%), and the following conditions each with 1 case and 0.27% frequency: abscess of corpus cavernosum and penis, congenital urinary meatus structure, hydronephrosis, retractile penis, scrotal pain, the second stage of orchidopexy, testicular mass, and webbed penis (Table 2).

**Table 2:** Urological indications with hyaluronic acid/alginate gel application

	Pre-operative Diagnosis	Frequency
Penoscrotal webbing	94	25.0%
Hidden penis	73	19.4%
Phimosis of penis	61	16.2%
Chordee	47	12.5%
Penile torsion	37	9.8%
Penile adhesions	16	4.3%
Hernia	15	4.0%
Undescended testicle	7	1.9%
Hypospadias	6	1.6%
Hydrocele in adults	3	0.80%
Split urinary stream	3	0.80%
Dysuria	2	0.53%
Peyronie's disease	2	0.53%
Retractile testis	2	0.72%
Abscess of corpus cavernosum and penis	1	0.27%
Congenital urinary meatus structure	1	0.27%
Hydronephrosis	1	0.27%
Retractile penis	1	0.27%
Scrotal pain	1	0.27%
Second stage of orchidopexy	1	0.27%
Testicular mass	1	0.27%
Webbed penis	1	0.27%
Total	376	100%

Postoperative outcomes

There were no associated postoperative complications associated with HA/alginate gel use. Out of the 142 male patients, no patients had any re-admission, re-operation, or subsequent complications at the 4-6 week follow-up.

## Discussion

This paper aimed to evaluate the intra-operative use of a novel hyaluronic acid/alginate hydrogel during urologic procedures to prevent postoperative tethering and improve surgical outcomes. The largest cohort and the only known urologic one published to date, our data demonstrate its successful application across a wide range of urologic procedures. The majority of patients in this study were pediatric patients (93.7%) and under 1 year of age (60.0%), with a total of 376 urologic diagnoses, most commonly, penoscrotal webbing, hidden penis, and phimosis of penis. There were no complications, re-admissions, or re-operations among the entire cohort, indicative of an excellent safety profile and overall excellent clinical outcomes at the four to six-week follow-up.

A key result of this study is the successful application of the HA/alginate hydrogel across a wide spectrum of urologic conditions [15], ranging from congenital anomalies, such as penoscrotal webbing and hidden penis, to acquired conditions like Peyronie’s disease and penile adhesions. While there is limited information on this novel HA/alginate hydrogel’s use during urology cases, it has been well characterized throughout a host of surgical environments [7,8,12]. The hydrogel’s adaptability to complex surgical environments, coupled with its biocompatibility and ease of application, makes it an attractive alternative to traditional anti-adhesion materials [16]. Unlike animal- or human-derived tissue products, the plant-based composition of the hydrogel eliminates risks associated with immunogenicity and disease transmission while maintaining effective bioresorption properties [10]. These advantages address many of the limitations encountered with earlier-generation anti-adhesion solutions such as incomplete resorption and inflammatory responses [5,17,18].

The study’s focus on pediatric patients (<1-year-old patients making up 60% of the cohort) provides important insights into the hydrogel’s utility in younger populations, where minimizing adhesions is critical for preserving anatomical integrity and function [19]. In surgeries for conditions like hidden penis, penoscrotal webbing, and phimosis, tissue mobility is vital for achieving optimal outcomes [20,21]. The absence of adhesion-related complications in this study suggests that the hydrogel effectively promotes healing while preventing the fibrous tethering of tissues that could compromise surgical success.

This study also highlights the hydrogel’s potential to improve surgical outcomes beyond urology. Its widespread use in other surgical disciplines, including orthopedics, plastic surgery, and neurosurgery, demonstrates its versatility in preventing adhesions and facilitating tissue gliding [12,13,17,18]. The study’s emphasis on urologic procedures expands this body of evidence, suggesting that the hydrogel can be particularly beneficial in surgeries where tissue mobility is paramount, such as those involving the penile shaft, neurovascular bundle, and other delicate anatomical structures.

This study has several potential limitations. As a retrospective, single-surgeon, single-center analysis, its generalizability is limited, as surgical expertise and patient populations can vary significantly across institutions. The study primarily included pediatric patients, with a majority under one year of age, which limits applicability to adult populations where tissue healing dynamics differ [22]. Additionally, the eight-week follow-up period may not capture long-term adhesion-related complications or outcomes, and the absence of a control group or direct comparison with other anti-adhesion products precludes definitive conclusions about the hydrogel’s relative efficacy. Subclinical adhesions or minor complications may have gone undetected without advanced imaging or functional assessments, and cost-effectiveness was not evaluated, which could influence its adoption in broader clinical practice. Future prospective, randomized studies with diverse patient populations, longer follow-up, and cost-benefit analyses are essential to validate these findings and establish the hydrogel’s role in urologic surgeries. 

While prior studies have examined limited use [23], future research should include prospective, randomized controlled trials to validate the findings of this study and further delineate the hydrogel’s efficacy compared to existing solutions. Investigating the hydrogel’s performance in more complex cases or adult populations could also provide valuable insights into its broader applicability. Moreover, studies examining the economic impact of using the hydrogel, such as reductions in re-operations, patient morbidity, and healthcare costs, would strengthen the argument for its widespread adoption. Nevertheless, overall, this paper is the first and largest case series to date, to the best of our knowledge, detailing the successful application of this novel HA/alginate hydrogel.

## Conclusions

This study highlights the promising role of a novel hyaluronic acid/alginate hydrogel (VersaWrap Nerve Protector, VersaWrap) in protecting peripheral nerves, preventing postoperative tethering, and thereby improving outcomes in pediatric and adult urologic surgeries. The data demonstrates VersaWrap’s effective application across a diverse range of urologic diagnoses, including penoscrotal webbing, hidden penis, and phimosis, with no reported complications, re-admissions, or re-operations during the follow-up period. These results emphasize the hydrogel’s excellent safety profile, biocompatibility, and functional efficacy in minimizing tissue tethering and enhancing healing. Given its versatility, ease of use, and adaptability to complex anatomical challenges, this novel HA/alginate hydrogel represents a significant advancement in adhesion prevention. Further research involving larger cohorts, diverse populations, and longer follow-up periods will be useful to confirm its long-term benefits and broader applications in surgical practice. This study paves the way for the integration of this hydrogel into routine urologic procedures, potentially improving patient outcomes and reducing the need for revision surgeries.
